# From climate perceptions to actions: A case study on coffee farms in Ethiopia

**DOI:** 10.1007/s13280-024-01990-0

**Published:** 2024-02-25

**Authors:** Xenia Gomm, Biruk Ayalew, Kristoffer Hylander, Francesco Zignol, Lowe Börjeson, Ayco J. M. Tack

**Affiliations:** 1 Department of Ecology, Environment and Plant Sciences, Stockholm University, 106 91 Stockholm, Sweden; 2 Department of Forest Ecology and Management, Swedish University of Agricultural Sciences, 901 83 Umeå, Sweden; 3 Department of Human Geography, Stockholm University, 106 91 Stockholm, Sweden

**Keywords:** Agroforestry, Climate adaptation, Climate change, Climate perception, Coffee, Ethiopia

## Abstract

Increasing temperatures and shifting precipitation patterns have major consequences for smallholder farmers, especially in the Global South. Our study examined spatial patterns and climatic drivers of farmers' perceptions of climate change, and how these perceptions translated into adaptation actions. We interviewed 56 farmers in southwestern Ethiopia and analyzed ERA5-Land reanalysis climate data from 1971 to 2020. The majority of farmers perceived the recorded temperature increase as well as a decrease and shift in the timing of rainfall. Perceived climate change varied with local climate factors and not with the rate of climate change itself. Farmers' adaptation practices showed associations with local temperature, but not with farmers’ perceptions of climate change. Our findings highlight that even if farmers perceive climate change, perceptions are most common in areas where climate action is already urgent, and perceptions may not translate into adaptation. Thus, targeted and timely information and extension programs are crucial.

**Supplementary Information:**

The online version contains supplementary material available at 10.1007/s13280-024-01990-0.

## Introduction

The global climate is rapidly changing, with increases in temperature, changes in precipitation patterns and increased climate variability (Stocker et al. 2013; UN.org 2021). Climate change is already having a major impact on global agriculture (Haggar and Schepp 2011). While several studies have pointed out that smallholder farmers in the Global South are particularly vulnerable (Morton 2007; Nelson et al. 2009), we lack insights in what aspects of climate change are perceived by smallholder farmers, and what factors can explain variation in perceptions among smallholder farmers living in different parts of the landscape. Understanding farmers’ perceptions of climate change is important, as perceptions play a major role in climate adaptation (Pauw 2013; Tesfaye et al. 2021). Overall, unravelling the link between climate change, farmers’ perceptions of climate change and adaptation measures will help to develop policies to support farmers with climate adaptation and make the landscape more climate-resilient.

From the perspective of climate adaptation, it is paramount that farmers accurately perceive changes in relevant climatic variables (Grothmann and Patt 2005; Pauw 2013; Tesfaye et al. 2021). For temperature, studies have shown that farmers’ perceptions of changes in temperature are generally consistent with climate records (Amadou et al. 2015; Roco et al. 2015; Habtemariam et al. 2016; Kibue et al. 2016; Tadesse et al. 2017; Uddin et al. 2017). In contrast, there is often a discrepancy between perceptions of, and meteorological changes in, precipitation. For example, Amadou et al. (2015) and Bryan et al. (2009) reported that smallholder farmers in Ghana, Ethiopia and South Africa perceived a reduction in precipitation, even though the meteorological data showed no such trend. Importantly, while the majority of studies on climate change perception have focused on the perception of changes in temperature, and some on precipitation, many other aspects of the climate are important for agriculture (Bhattacharya 2019). Examples of critically important, but often ignored, climatic events are the occurrence and duration of cold and hot spells, unseasonal rain and the frequency of droughts (Massetti and Mendelsohn 2015; Bhattacharya 2019; Torres et al. 2019; Orimoloye et al. 2022).

Perceptions of climatic changes can vary strongly among farmers living in different parts of the landscape, and understanding the causes of this variation is crucial for targeted information programs and developing policies for climate adaptation (Arbuckle et al. 2013). One cause of the spatial variation in climate perceptions might be spatial variation in the current climate (for example, variation between farms in the 1991–2020 mean temperature and precipitation, also referred to as climate normals). Another cause of spatial variation in climate perceptions might be caused by spatial variation in the actual rate of climate change (e.g., how temperature and precipitation change between 1991 and 2020 varied between farms). Despite this, we found only one study targeting the relationship between spatial variation in climate perception and current climate (Amadou et al. 2015), and we are not aware of any other study that investigated the relationship between spatial variation in climate perception and the actual rate of climate change. Overall, we lack knowledge of whether spatial variability in farmers' perceptions of climate change is related to spatial gradients in current climate or spatial gradients in the rate of climate change.

While adaptation to climatic changes is crucially important for food security (Nelson et al. 2009; Adhikari et al. 2015), we lack fundamental insights necessary to efficiently initiate, facilitate or otherwise support such adaptations. Two questions that urgently need to be answered are: (i) Will perceptions of climate change autonomously be translated into adaptation, or is there a need for support by agricultural extension officers and policy-makers? and (ii) Will adaptation processes be uniform across the landscape, or are they influenced by local climatic conditions? In short, we need to understand how perceptions of climate change, as combined with local climate, jointly shape adaptation.

Arabica coffee (*Coffea arabica *L.) is an important agricultural commodity globally and in Ethiopia. On a global scale, coffee is the primary source of income for 125 million people (UNCTAD 2003). Originating from southwestern Ethiopia, Arabica coffee plays a crucial role in the Ethiopian economy, accounting for 34% of export earnings and providing livelihoods for approximately 15 million Ethiopians (Davis et al. 2012; Moat et al. 2017). The coffee industry in Ethiopia is currently encountering significant challenges as a result of climate change (Davis et al. 2012; Moat et al. 2017). Given the high sensitivity of coffee to changing climatic conditions, there is an urgent need for adaptation measures to minimize the potential losses faced by coffee farmers.

**Fig. 1:**
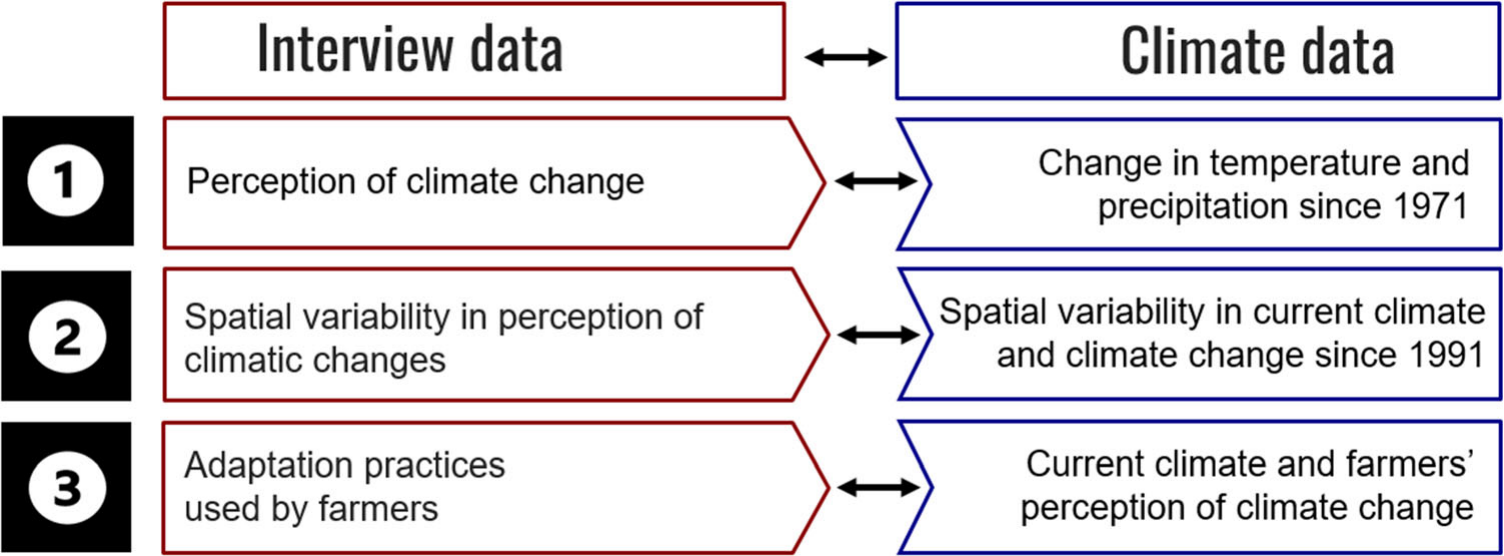
Conceptual framework linking farmers’ perceptions and actions with climatological records. For each of three questions, we linked the interview data (left, red) and climate data (right, blue): (1) What is the relationship between farmers’ perceptions of climate change and historical climate data? (2) Is the spatial variability in farmers’ perceptions of climate change related to spatial gradients in the current climate or spatial gradients in the rate of climate change? and (3) Which adaptation practices did farmers use to meet challenges due to climate change and are these adaptation practices related to spatial gradients in the current climate or farmers’ perceptions of climate change?

The overarching aim of this study was to examine the relationship between climate change, farmers’ perceptions of climate change and management adaptation. For this, we interviewed 56 coffee farmers and analyzed historical climate data from the ERA5-Land reanalysis dataset for the period 1971–2020. More specifically, we addressed the following questions:

1. What is the relationship between farmers’ perceptions of climate change and historical climate data? (Fig. 1).
2. Is the spatial variability in farmers’ perceptions of climate change related to spatial gradients in the current climate or spatial gradients in the rate of climate change? (Fig. 1).
3. Which adaptation practices did farmers use to meet challenges due to climate change and are these adaptation practices related to spatial gradients in the current climate or farmers’ perceptions of climate change? (Fig. 1).

## Materials and methods

### Study area

**Fig. 2:**
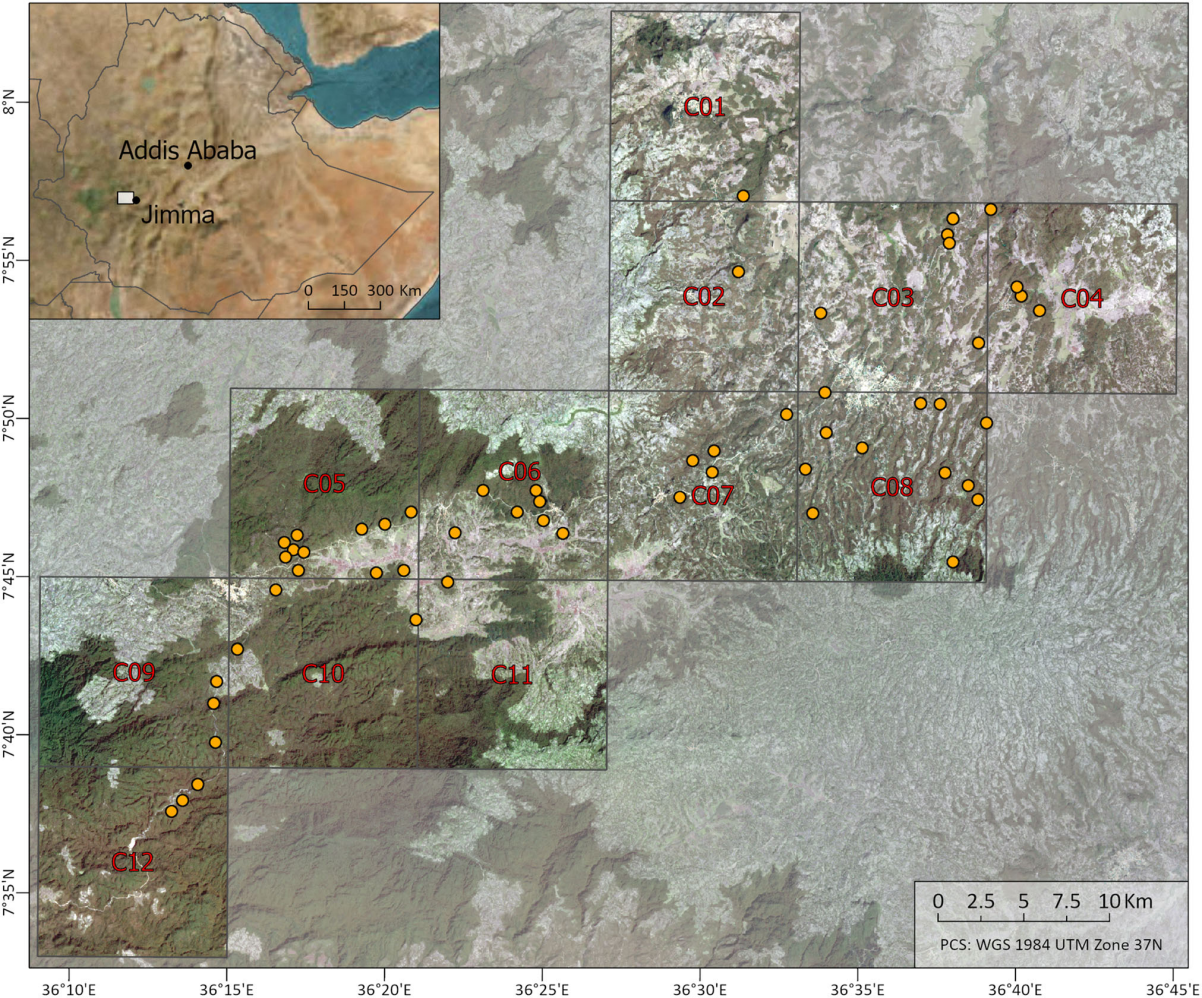
Location of the 56 study sites in the Gera and Gomma districts in Jimma zone, southwestern Ethiopia. The inset shows the location of the study region within southwestern Ethiopia, the yellow circles on the main map represent the study sites and the squares the ERA5-Land grid cells

The study area is located in the Gomma and Gera districts (7° 37′–7° 57′ N and 36° 13′–36° 40′ E) of Jimma zone in Oromia National Regional State in southwestern Ethiopia (Fig. 2). The average daily minimum and maximum temperatures are 12 °C and 28 °C, respectively, with only little seasonal fluctuations (Zignol et al. 2023). The annual precipitation typically varies between 1500 and 2100 mm (Zignol et al. 2023). The amount of precipitation varies greatly within a year, with a dry season from November to April and a rainy season from May to October. The landscape is characterized by a mosaic of larger Afromontane moist forests, fragmented forest patches, grazing lands and agricultural fields (Zewdie et al. 2020).

Coffee is grown within the natural forest, smaller forest fragments and forest edges (Zewdie et al. 2020). Temperature and rainfall are the two most important factors influencing coffee plant growth (Haggar and Schepp 2011). Even slight deviations in temperature and rainfall can cause plant death or decreased plant performance, which results in specific requirements for its growth (Haggar and Schepp 2011; Iscaro 2014; Bunn et al. 2015). Temperatures slightly below 0 °C can only be survived by Arabica coffee plants for a very short period of time between minutes and a couple of hours (Davis et al. 2019). High temperature above 32 °C can be survived by Arabica coffee depending on the soil humidity (Davis et al. 2019). The pattern of rainfall is important for coffee production (Camargo 2010). The isolated rain showers that characterize the end of the dry season initiate the flowering period of coffee. Coffee berries develop during the rainy season and are harvested at the beginning of the dry season. In this region, the income of 77% of smallholder farmers depends on coffee production, and the impact of climate change on coffee production will thus affect millions of lives (Diro et al. 2019).

### Site selection

Within the study area, 60 sites with coffee production were selected by Zewdie et al. (2020), of which we considered 56 sites due to data availability (Fig. 2). The study sites were chosen to encompass a wide gradient of coffee management ranging from little managed coffee grown in the natural forest to coffee grown in commercial plantations. The study sites ranged in altitude from 1506 to 2159 m a.s.l. For more details, see Zewdie et al. (2020).

### Interviews

To understand the relationship between climate change, farmers’ perceptions of climate change and their adaptation practices, we interviewed 48 smallholder farmers and eight sub-managers at commercial plantations (henceforth collectively referred to as ‘farmers’). Climate adaptation was thereby defined as adjustments made to reduce potential damages associated with climate change. The interviews, which contained both closed- and open-ended questions, were conducted between February and March 2020 in Amharic or Afaan Oromo, the local languages in the study area. Most of the interviews were conducted on the coffee farm itself. The interviews contained a rich set of questions on climate change, perception of climate change, pests and diseases and coffee management practices (see Text S1). Regarding questions on climate change, farmers were asked how different climate variables had changed, including the dry season temperature, rainy season temperature, length of hot spells, length of cold spells, the frequency of cold nights, the quantity of rainfall during the rainy season, length of the rainy season, length of the dry spells during the rainy season, unseasonal rain in the dry season, intensity of rainfall and the frequency of droughts. We used the year 1991 as a historical reference point for the questions, as this is (i) the year of the fall of the Derg regime (which all farmers remembered well), and (ii) the 30-year period from 1991 to 2021 matches the period across which climate normals are calculated. While we also asked farmers in an open question whether they perceived nonlinear patterns of climate change, referring back to well-known historical reference points, including the rule of Haile Selassie (c. 1970), fall of the Derg (1990), leadership of Meles Zenawi (c. 1991–2012) and the election of Abiy Ahmed (2018), farmers consistently reported increases, decreases or no change. Hence, for the analyses, we use the three categories 'Increase,’ ‘Decrease’ or ‘No change.’ Regarding questions on adaptations to climate change, farmers were asked whether they had adapted management practices in response to changes in temperature, precipitation and drought, which included soil and water conservation, shade management, small-scale irrigation, use of improved coffee varieties, fertilizer application, organic matter application, pesticides application, disease and pest management, mulching, intercropping, crop diversification, livestock rearing, off-farm labor, shifting coffee to another crop and relocation of coffee to a more suitable area.

### Climatic and environmental data

To link farmers’ perceptions to climate data, we used time series from the land component of the fifth generation of European ReAnalysis (ERA5-Land; Muñoz Sabater 2019) produced by the European Centre for Medium-Range Weather Forecasts (ECMWF). This dataset covers the study area with a spatial resolution of 11.1 km per grid cell from 1950 onwards (Fig. 2). We focused on two periods: (i) the 50-year period 1971–2020 and (ii) the 30-year period 1991–2020. We extracted temperature (at 2 m above the surface, ºC) and total precipitation (in mm) on annual and monthly timescales. Monthly data were averaged separately for the dry season from November to April and for the rainy season from May to October. The occurrence and intensity of droughts were analyzed by the Standardized Precipitation Evapotranspiration Index (SPEI) (McKee et al. 1993).

### Statistical analyses

All statistical analyses were conducted in R v. 4.1.2 (R Core Team, 2020).

*Climate change and farmers’ perceptions of climate change—*To analyze temporal changes in temperature, precipitation and drought, we used the Mann–Kendall trend test (Mann 1945). For a dataset of *N* = 30 and *N* = 50 years, the Mann–Kendall trend is significant at $\alpha <0.05$ if $\tau \ge |0.255|$ and $\tau \ge |0.192|$, respectively. As an estimate of the magnitude of change, we used Sen’s slope (Sen 1968). To explore farmers’ perceptions of changes in temperature, precipitation and droughts, we used Chi-square goodness-of-fit tests as implemented in the *chisq.test* function in the base R package (R Core Team, 2020). Given the limited number of farmers who indicated ‘no change’ (see ‘Discussion’), we conducted the test using two categories (increase and decrease), where we assumed an equal probability of outcomes (i.e., 50:50) under the null hypothesis.

*Drivers of spatial variation in farmers’ perceptions—*To examine the relationships between farmers’ perceptions of climate change and spatial variability in local climatic variables, we used the framework of generalized linear models. We modeled perceptions of climate change as a function of climate data (temperature, precipitation and SPEI), with a binary distribution and logit link. We used similar models to examine the relationship between farmers’ perceptions and spatial variability *in the rate* of temperature change and rate of change in drought severity, where we used the Sen’s slope of temperature and the Sen’s slope of drought severity as the predictor variables. As the temporal trend in precipitation was not significant (see ‘Results’), we did not include analysis of farmers’ perceptions and spatial variability *in the rate* of precipitation change. For the full set of relationships tested, see Tables S1 and S2. To avoid problems with collinearity and overfitting, we used simple regression models instead of multiple regressions. Models had 56 replicates (i.e., farmers), allowing for the detection of medium to weak effect sizes (Harrell et al. 1984).

*Relationship between farmers’ perceptions and adaptations—*To explore the influence of spatial variation in temperature, precipitation and drought, as well as the perception of temperature and precipitation changes, on adaptation practices at the different study sites, we used PERMANOVA implemented in the function *adonis2* in the *vegan* package (Oksanen et al. 2020). Because the adaptation practices were coded as binary variables (i.e., presence–absence of a set of management practices), the Jaccard metric was used to create the dissimilarity matrix as based on the matrix of adaptation practices by location. We then modeled the composition of adaptation practices as a function of spatial variation in local temperature, precipitation and droughts, as well as farmers’ perception of climatic changes. We visualized the patterns and drivers with non-metric multidimensional scaling (NMDS) of the site-by-adaptation matrix, as implemented in the function *metaMDS* in the R package *vegan* (Oksanen et al. 2020).

## Results

### Relationship between climate change and farmers’ perceptions

**Fig. 3:**
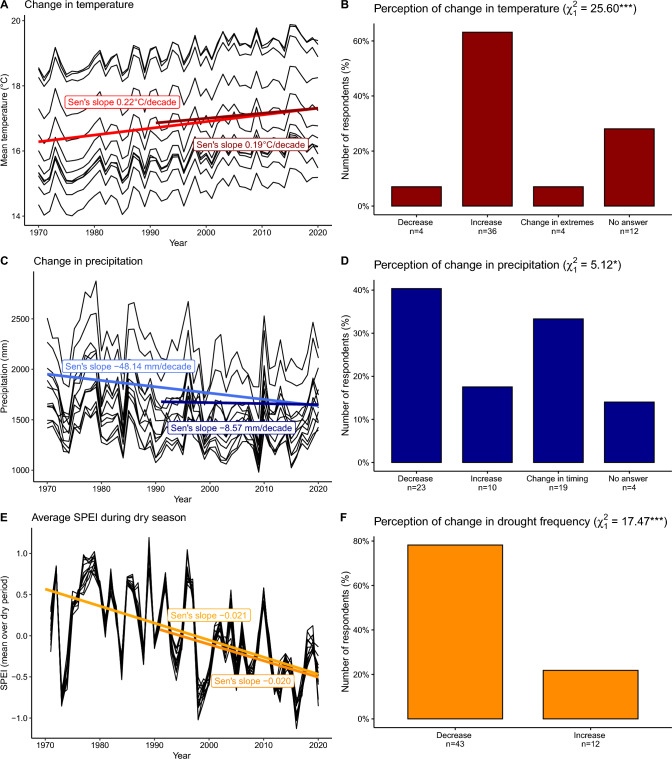
Climate change and farmers’ perceptions of climate change in Jimma zone in southwestern Ethiopia. Panels **A**, **C** and **E** show the mean temperature (°C), average annual precipitation (mm) and SPEI from 1971 to 2020 for the study area. The dark and light thick lines represent the Sen’s slope of the average temperature, precipitation and SPEI in the study area for a 30- and 50-year period, respectively. The thin black lines represent the individual ERA5-Land grid cells in our study area. For SPEI, negative values represent dry periods. Panels **B**, **D** and **F** visualize answers of farmers to the question of how temperature, precipitation and frequency of droughts have changed during the past 30 years. Given in parenthesis are the *X*^2^-value and significance (***p < 0.001, *p* < 0.01, *p < 0.05) of the difference between farmers reporting an increase or decrease in a climatic variable

The annual mean temperature in the study area increased significantly during the periods 1971–2020 (Kendall’s $\tau = 0.611$, Sen’s slope = 0.22 °C/decade) and 1991–2020 (Kendall’s $\tau= 0.439$, Sen’s slope = 0.19 °C/decade; Fig. 3A), amounting to a temperature increase of c. 1.0–1.1 °C within the past 50 years. The increase in temperature was experienced by 64% of the interviewed farmers (Fig. 3B). The average precipitation decreased significantly during the period 1971–2020 (Kendall’s $\tau=-0.290$, Sen’s slope = − 48.14 mm/decade), but showed no significant trend for the past 30 years (Kendall’s $\tau=-0.048$,  Sen’s slope = − 8.57 mm/decade, Fig. 3C). The answers of farmers to the open-ended question about changes in precipitation over the past 30 years were variable: 41% of the farmers stated that they had observed a decrease in precipitation, 18% answered that they had noticed an increase in precipitation and 34% reported a change in the timing of precipitation (Fig. 3D). While climate data showed clear evidence for increased drought during the periods 1971–2020 (Kendall’s $\tau = -0.373$,  Sen’s slope = − 0.21/decade, Fig. 3E) and during the periods 1991–2020 (Kendall’s $\tau=-0.287$ , Sen’s slope = − 0.20/decade, Fig. 3E), 78% of the farmers indicated that the frequency of droughts decreased, and only 22% reported that droughts increased during the past 30 years (Fig. 3F).

**Fig. 4:**
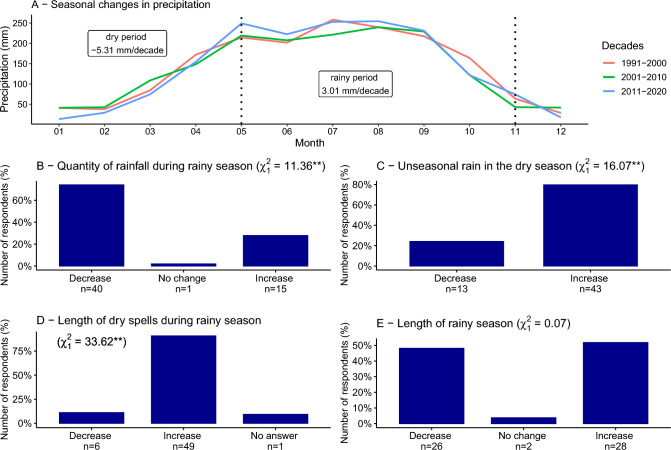
Seasonal changes in precipitation in the study area in Jimma zone in southwestern Ethiopia. Panel **A** shows the seasonal distribution in precipitation, separately for the years 1991–2000, 2001–2010 and 2011–2020, as calculated from the ERA5-Land reanalysis dataset. The vertical bar plots in panels **B**–**E** present the answers of 56 farmers who have been asked about precipitation changes during the past 30 years. Given in parenthesis are the *X*^2^-value and significance (**p < 0.01, *p < 0.05) of the difference between farmers reporting an increase or decrease in a climatic variable

Regarding the shift in timing of rainfall during the past 30 years, climate data showed that rainfall decreased during the dry season (5.3 mm per decade) and increased during the rainy season (3.0 mm per decade; Fig. 4A). The majority of farmers perceived a decrease in the quantity of precipitation during the rainy season (Fig. 4B, χ_1_^2^ = 11.36, *p* < 0.001), increase in unseasonal rain during the dry period (Fig. 4C, χ_1_^2^ = 16.07, *p* < 0.001) and increase in the length of dry spells during the rainy season (Fig. 4D, χ_1_^2^ = 33.62, *p* < 0.001). When asked about the development of the length of the rainy season, there was no clear majority for any answer: 50% of respondents perceived an increase in the length of the rainy season, while 46% perceived a decrease in the length of the rainy season (Fig. 4E, χ_1_^2^ = 0.07, *p* = 0.785).

The mean temperature between 1991 and 2020 increased both during the dry (0.28 °C per decade) and rainy seasons (0.13 °C per decade; Fig. S1). Matching this pattern, the majority of farmers perceived an increase in the dry (75%, Fig. S1B, χ_1_^2^ = 14.00, *p* < 0.001) and rainy season temperature (64%, Fig. S1C, χ_1_^2^ = 5.30, *p* = 0.021), and longer hot (71%, Fig. S1D, χ_1_^2^ = 11.36, *p* < 0.001) and shorter cold spells (63%, Fig. S1E, χ_1_^2^ = 3.50, *p* = 0.061).

### Drivers of spatial variation in farmers’ perceptions

The annual mean temperature and precipitation were highly variable across the study area (Figs. S2 and S3). Temperature increased from 14 to 21 °C from the southwest to northeast, whereas precipitation decreased from 3000 to 1000 mm along the same gradient (Figs. S2 and S3). The dry period increased from the southwest to northeast (Fig. S4).

**Fig. 5:**
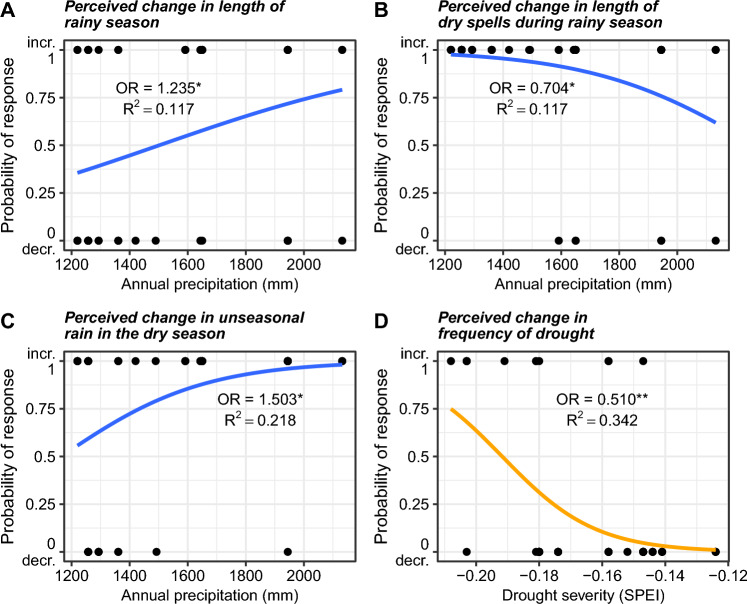
The relationship between farmers’ perceptions of climatic changes and local climate in Jimma zone in southwestern Ethiopia. Shown are the relationships between perceived changes in climatic variables during the past 30 years and spatial variation in **A**–**C** annual precipitation and **D** drought severity. The data points represent whether the farmer perceived an increase or decrease in the climatic variable, and the trend line shows the fitted relationship between local climate and farmers’ perceptions of climate change. Given in text are the odds ratio (OR, with 1 °C, 100 mm and 0.01 in SPEI as one unit, respectively), significance (**p < 0.01, *p < 0.05) and R^2^. For the full set of relationships tested, see Table S1

The perception of changes in dry and rainy season temperature, length of hot and cold spells and the frequency of frost or cold nights were not significantly related to spatial variation in annual mean temperature (Table S1). Farmers who lived at locations with higher annual precipitation were more likely to perceive an increase in the length of the rainy season (Fig. 5A), a decrease in length of dry spells during the rainy season (Fig. 5B) and an increase in the frequency of unseasonal rain during the dry season (Fig. 5C). The perception of changes in the quantity and intensity of rainfall during the rainy period were not significantly related to spatial variation in annual precipitation (Table S1). Further, more severe droughts were not significantly related to the perception of unseasonal rain in the dry season, dry season temperature, length of hot and cold spells and the frequency of cold nights (Table S1). Farmers who lived in locations with more severe droughts (i.e., lower SPEI index) were more likely to perceive an increase in drought frequency (Fig. 5D).

The rate of climate change in terms of temperature during the past 30 years varied across the study sites between 0.15 °C per decade and 0.22 °C per decade (Fig. S5). Spatial variation in the rate of changes in drought over the past 30 years was apparent when Walter diagrams of different locations are compared (Figs. S6 and S7, Table S3). The perception of changes in dry and rainy season temperature, length of hot and cold spells and the frequency of frost or cold nights were not significantly related to spatial variation in the rate of temperature change (Table S2). The perception of changes in unseasonal rain in the dry season, dry season temperature, length of hot and cold spells and the frequency of cold nights did not correlate significantly with the Sen’s slope of drought severity (Table S2).

### Relationship between farmers’ perceptions and adaptations

**Fig. 6:**
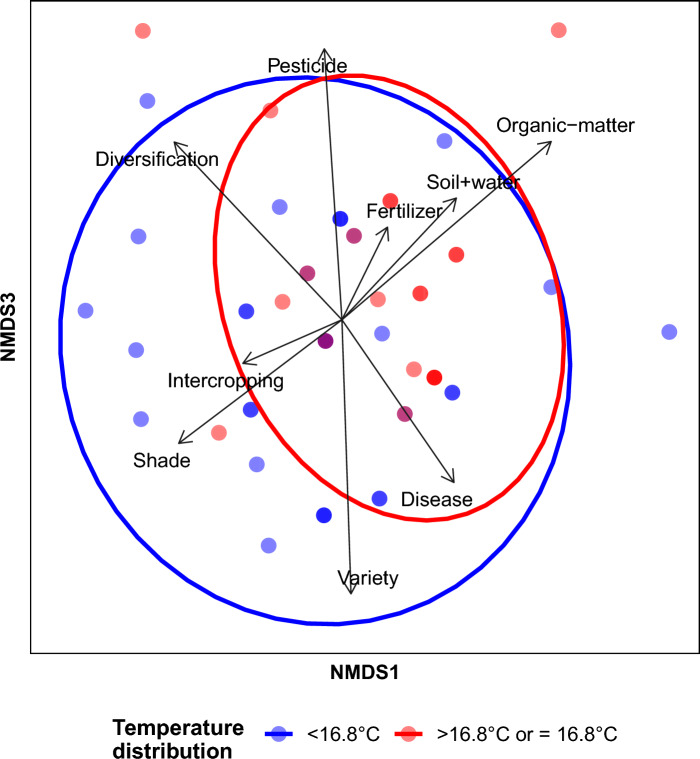
Relationship between the composition of adaptation practices and temperature distribution. The vector names represent the different adaptation practices. The circles indicate the temperature distribution below (blue) and above (red) the mean temperature across all study sites of 16.8 °C. When two blue data points overlap this results in a dark blue circle, when two red data points overlap this results in a dark red circle and when one blue and one red data point overlap this results in a purple circle. The stress value is 0.102 (Clarke 1993). In the figure, only axis one and three of the three-dimensional ordination are shown, as they most clearly showed the differentiation between the two temperature groups. For pairwise plots of all three dimensions, see Fig. S8

Farmers’ adaptation actions were related to spatial variation in temperature, but not to spatial variation in precipitation, drought severity or the perceptions of change in temperature or precipitation (Fig. 6, Table S4). There is a complex pattern of variation in adaptation actions among the farmers, but it seems like farmers in locations with warmer temperatures more often apply organic matter and do soil-and-water conservation practices, while farmers at colder locations more frequently diversified their crop, managed the shade levels and improved the coffee variety (Fig. 6).

## Discussion

By combining interviews and historical and present climate data, we examined the relationship between climate change, farmers' perceptions thereof and the implications for adaptation practices. The majority of farmers perceived the recorded temperature increase, as well as a decrease and shift in the timing of rainfall. Climate change perceptions were related to local climatic variables, but not to spatial variation in the rate of climate change. The composition of farmers’ adaptation practices showed a weak association with local temperature, but there was no effect of the perception of climate change on adaptation practices. Taken together, our findings emphasize that (i) changes in precipitation are equally or even more important than changes in temperature, (ii) spatial variation in the current climate matters for farmers’ perceptions of climate change and (iii) farmers’ perceptions are not necessarily translated into actions.

As expected, the majority of farmers perceived the increase in temperature during the past three decades, which matches findings from Ethiopia (Esayas et al. 2019; Habtemariam et al. 2016; Tadesse et al. 2017; Tesfaye et al. 2021; Weldegebriel and Prowse 2017) and other parts of the world, such as Bangladesh (Uddin et al. 2017) and Chile (Roco et al. 2015). While it is hard to match the climate data to the climatic niche of Arabica coffee, as the physiology, growth and yield of Arabica coffee depend on so many factors, we may note that the mean annual maximum temperature varied between 20 and 27 °C across the study area, and thus did not exceed the ideal average maximum temperature of Arabica coffee of 25–27 °C (Davis et al. 2019). Regarding long-term trends in precipitation, the two most common answers were a decrease in rainfall or a shift in the timing of rainfall. While we did not detect a significant decrease in rainfall during the past 30 years, the common perception of a decrease in rainfall can likely be explained by the fact that rainfall did decrease since the 1970s and 1980s in the Ethiopian Highlands, as it is for many areas in East Africa (Giannini et al. 2008; Williams & Funk 2011). That a temporal shift in rainfall is perceived by the farmers is a phenomenon reported in several other studies (Amadou et al. 2015; Esayas et al. 2019; Tesfaye et al. 2021). The sensitivity of farmers toward temporal shifts in rain might be explained by the fact that coffee is very sensitive to the presence or absence of precipitation during specific periods, such as the requirement of early rain to initiate flowering. Although drought (as measured by SPEI) increased during the past decades, 78% of farmers perceived a decrease in the frequency of droughts. Here, the definition of drought by individual farmers might play an important role, as this definition depends on the socio-economic situation of individual families (Meze-Hausken 2004) and on past experiences (Slegers 2008). This is especially important considering the numerous droughts and famines Ethiopia has already experienced, with the most severe droughts occurring between 1984 and 1988 (El Kenawy et al. 2016). Knowledge or experience of severe droughts prior to 1991 may explain why 78% of farmers reported a decrease in drought frequency, even though drought as defined in this study has increased over the past 30 years.

Our interviews revealed large spatial variation in the perception of changes in temperature, precipitation and drought. While this variability could not be explained by spatial variation in the rate of climate change, it was correlated to spatial variation in the local climate. Two key findings emerged from this: (i) From the local climatic factors, precipitation was more important than temperature in shaping farmers’ perceptions of climate change, and (ii) changes in a particular climatic variable were perceived more clearly when that particular climatic variable was already a limiting factor, which might be due to a threshold effect. For example, farmers were more likely to perceive an increase in dry spells when local precipitation was relatively low. Yet, this contrasts with studies from Deressa et al. (2008) and Amadou et al. (2015), who reported that farmers living in the Ethiopian and Ghanaian highlands were more likely to have a good perception of climate change than farmers in the lowlands. Since our study demonstrated a general trend of climatic changes being perceived more strongly in areas where they already had a greater impact on coffee yields, this could mean that the accurate perception of climatic changes will increase in the future. Notably, the absence of a relationship between the perception of climate change and the rate of climate change might be due to the fact that the rate of climate change is relatively uniform across the study landscape (e.g., Fig. S5).

Adaptation was not related to the perception of climate change, but showed a weak relationship with local temperature. The absence of an effect of the perception of climate change on adaptation actions matches the findings of several studies that perceptions per se do not necessarily translate into adaptation (Pauw 2013; Tesfaye et al. 2021). One reason for the lack of a relationship between perceptions and actions in our study might be that implementing adaptation strategies is a time-intensive and expensive process making it especially for smallholder farmers difficult to engage in. Regarding the local climate, farmers at locations with higher temperature were more likely to apply organic matter and adopt soil and water conservation, which are both important measures for maintaining moist, productive soils in hotter and dryer environments (Delgado et al. 2011; Pritchard 2011). Overall, the adaptation actions reported in our study match those in other coffee growing areas, such as Latin America, where the dominant adaptations taken by farmers are the planting of trees, applying pesticides, adopting soil and water conservation practices and using more fertilizer (Harvey et al. 2018). While soil and water conservation are commonly applied adaptation actions also in other regions (Bryan et al. 2009), Howden et al. (2007) caution that while soil moisture conservation and water management have substantial potential for adaptation to climate change impacts, most of the benefits level off at temperature increases above 2 °C. While we explored the effects of climatic variables and perceptions thereof on climate change adaptation, many management adaptation practices are also influenced by social and economic factors (Pauw 2013; Tesfaye et al. 2021), which were not considered in the current study, and might explain the relatively low amount of variation explained in some of the analyses. Moreover, while we explicitly asked for management adaptations in response to climate change, some of the management adaptations might have been taken not only in response to climate change, but also for other reasons, such as higher yields or disease control.

### Implications

Our findings on the relationship between climate change, farmers' perceptions of climate change and adaptation measures are relevant for local agricultural extension officers and policy-makers, as well as for scientists and policy-makers worldwide.

For local agricultural extension officers and policy-makers, our findings highlight the need to tailor their support to farmers' individual situations, as farmers face different challenges due to the spatial variability of the local climate. Based on our findings, three recommendations can be derived: (i) Farmers living in locations with lower annual rainfall should be given special support to adapt to dry spells during the rainy season, e.g., through better water management systems, (ii) farmers living in locations with higher annual rainfall should be supported to cope with unseasonal rainfall during the dry season (as coffee needs a drier period to develop the flower bud, and this process can be halted by unseasonal rainfall) and (iii) farmers in locations with more severe drought should be supported to adapt to these circumstances, e.g., by replacing coffee shrubs with more heat-resistant varieties. Since some farmers are financially dependent on the crop and, therefore, cannot necessarily plan years in advance, policy-makers need to look at the long-term changes.

For global scientists and policy-makers, we would like to highlight three key messages. First, even though most scientific studies squarely focus on mean increases in temperature, changes in precipitation patterns and extreme weather events, such as cold and hot spells, are equally, or more important, for farmer’s perceptions and yield. Hence, climate modeling, interviews and adaptation studies should focus on the full set of relevant climatic variables. Second, our findings emphasize that farmers perceive specific trends more clearly when they become a limiting factor, which implies that farmers might perceive changes only when it is too late, or more difficult, to adapt. The question of timing might be particularly problematic within agroforestry systems with perennial crops, where adaptation measures might take more time to implement. Third, perceptions of climate change do not autonomously translate into adaptation actions, stressing the need to investigate which farmers to incentivize and support at what point in time. Importantly, even if farmers perceive climate change, those perceptions might be incomplete, too late and result in no or only partial management adaptation. Thus, timely and targeted information and support programs are crucial.

### Supplementary Information

Below is the link to the electronic supplementary material.

Supplementary file1 (PDF 1652 kb)
