# Supplementary material for: From climate perceptions to actions: A case study on coffee farms in Ethiopia
Source: Ambio. 2024 Feb 25;53(7):1002–14. doi: 10.1007/s13280-024-01990-0 (PMC11101399; doi:10.1007/s13280-024-01990-0)
Supplement: Supplementary file 1 — Supplementary file1 (PDF 1652 kb) [file 13280_2024_1990_MOESM1_ESM.pdf]

Supplementary materials

From climate perceptions to actions: A case study on coffee farms in Ethiopia

Xenia Gomm[1 *], Biruk Ayalew[1], Kristoffer Hylander[1], Francesco Zignol[1], Lowe Börjeson[2] and Ayco J. M. Tack[1]

[1] Department of Ecology, Environment and Plant Sciences, Stockholm University, Svante Arrhenius väg 20A, SE-114 18 Stockholm, Sweden.

[2] Department of Human Geography, Stockholm University, Svante Arrhenius väg 8, SE-114 18 Stockholm, Sweden.

*Corresponding author

Word count (including references): 5.814

Xenia Gomm,	MSc at the Bolin Center<br>Stockholm University, SE-106 91 Stockholm, Sweden<br>Accounting and Climate Policy Officer,<br>GermanZero e.V., 10587 Berlin, Germany<br>gomm.xenia@gmail.com, +49 17682564604
Biruk Ayalew,	PhD student at the Department of Ecology, Environment and Plant Sciences,<br>Stockholm University, SE-106 91 Stockholm, Sweden<br>biruk.nurihun@su.se

Kristoffer Hylander, Head of Department of Ecology, Environment and Plant Sciences, Stockholm University, SE-106 91Stockholm, Sweden

kristoffer.hylander@su.se

Francesco Zignol, Postdoctoral researcher at the Department of Forest Ecology and Management, Swedish University of Agricultural Sciences, 901 83 Umeå, Sweden

francesco.zignol@slu.se

Lowe Börjeson, Associate professor at the Department of Human Geography, Stockholm University, SE-106 91 Stockholm, Sweden

lowe.borjeson@humangeo.su.se

Ayco Tack, Professor at the Department of Ecology, Environment and Plant Sciences, Stockholm University, SE-106 91 Stockholm, Sweden

ayco.tack@su.se

Text S1: Questionnaire

Only the extract of the questionnaire used in the analysis of this study is presented below. The complete questionnaire is longer and includes also questions related to coffee diseases.

Section A, part 3

1. Have you personally noticed that the weather has changed since the fall of the Derg?

a. Yes

b. No

2. If so, how do you think the weather/climate has changed during the last 30 years? (In terms of temperature, rainfall etc.)

3. Generally, how do you think the following weather variables have changed during the last 30 years? Please place an "X" at the best answer.

**Weather Variable**	**Increase**	**No change**	**Decrease**	**Don't know**	**Remark**
**Rainfall**					
A. Quantity of rainfall during rainy season					
B. Length of rainy season					
C. Timing of rainy season (if it's changed how?) *					
D. Length of dry spells during rainy season					
E. Unseasonal rain in the dry season					
F. Intensity/ heaviness of rain					
**Temperature**					
A. Dry season temperature					
B. Rainy season temperature					
C. Length of hot spells					
D. Length of cold spells					
Frequency of frost / cold night					
Frequency of drought					

Section A, part 4

If you have observed changes in the climate, how has this impacted your farm?

1. How did it affect yield?
2. Is there any other effect of climate on your coffee farm?
3. In your opinion, how informed are you about climate change?
    a. Very well informed ("I know everything about climate change")
    b. Informed ("I know the basics of future climate change")
    c. Not very well informed ("I have heard about climate change before")
    d. Not informed at all ("no idea what climate change entails")

<u>Section B, part 1</u>

1. How did you change your management practice since the fall of the Derg? Why?
2. Have you changed your input use (fertilizer, herbicide)? Why?
3. Have you changed the varieties you plant? Why?
4. Have you managed the shade trees (such as number and choice of trees, shade regulation i.e., thinning or planting trees)?
5. Do you intercrop any other crops or trees in your coffer these days? A. Yes B. No
    a. If yes: what crops / trees?
    b. For what reason do you intercrop them?
    c. If no: why not?
6. Have you changed any other aspects of your management?
7. Have any other management changes been done in response to high temperature and shortage of rainfall? A. High temperature B. Shortage of rainfall?

8. Have you used the following management practice for your farm in response to changes in rainfall and temperature? Please place an “X” at the best answer

R/N	Adaptation strategy option	Yes	No	Why? Why not? Is the farmer planning to?
**1**	Soil and water conservation			
**2**	Shade tree planting*			
**3**	Small scale irrigation			
**4**	Improved coffee variety			
**5**	Fertilizer application			
**6**	Organic matter application			
**7**	Pesticides application			
**8**	Disease and pest management			
**9**	Use mulching to adapt to drought			
**10**	Intercropping			
**11**	Crop diversification to reduce risks			
**12**	Livestock rearing			
**13**	Off-farm labor/ income			
**14**	Shifting the coffee to another crop			
**15**	Relocation of coffee to more suitable area			

* Farmers were asked about "planting shade trees," but the responses and interpretation of this question referred to the same practices listed under the category of "shade management". For simplicity and to reduce reader confusion by using two different terms for the same practice, I have referred to this question as "shade management" in this study.

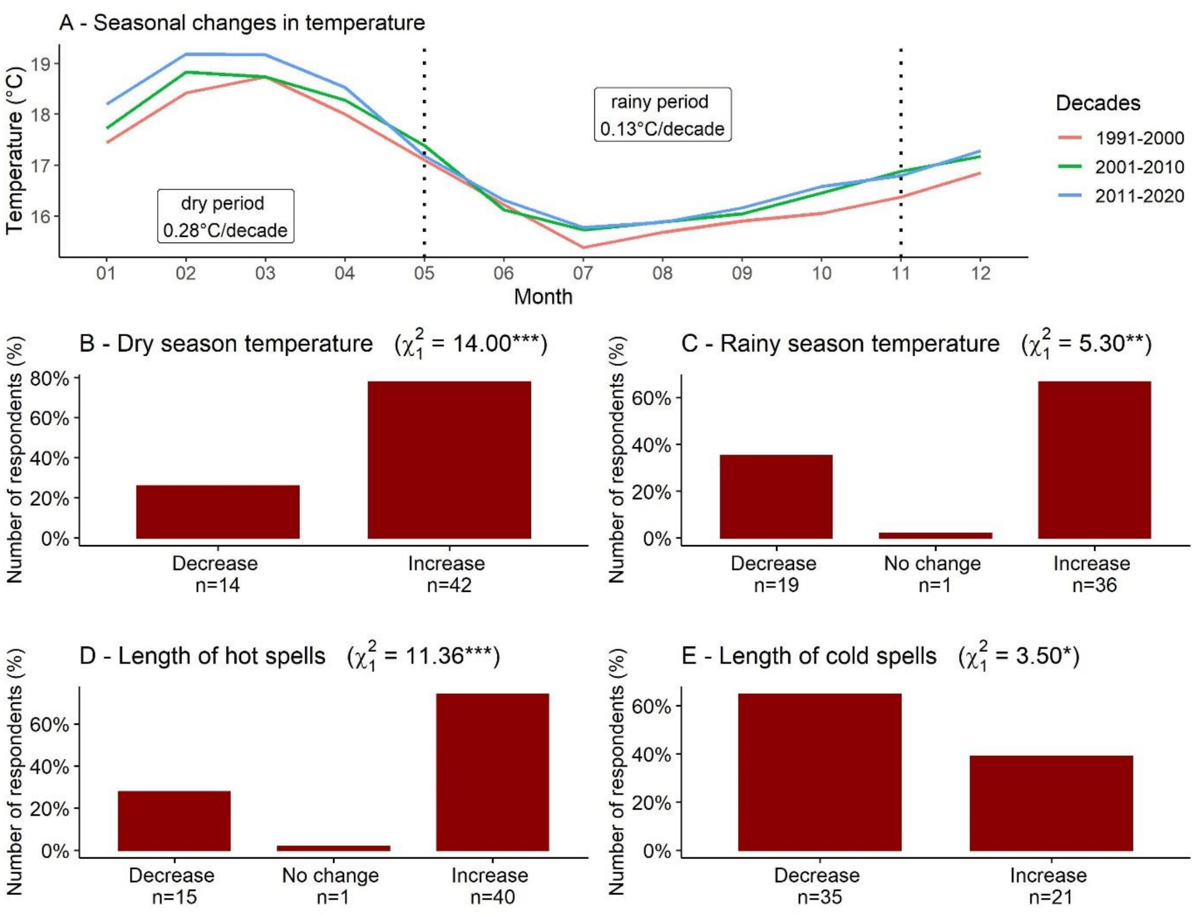

Figure S1: Seasonal changes in temperature in the Jimma zone in southwestern Ethiopia. Panel **A** shows the seasonal distribution of temperature, separately for the years 1991-2000, 2001-2010 and 2011-2020, as calculated from the ERA5-Land reanalysis dataset The vertical bar plots in panels **B** to **E** present the answers of 56 farmers who have been asked about temperature changes during the last 30 years. Given in parenthesis are the X^2-value and significance (*** $p < 0.01$, ** $p < 0.05$, * $p < 0.1$) of the difference between farmers reporting an increase or decrease in a climatic variable.

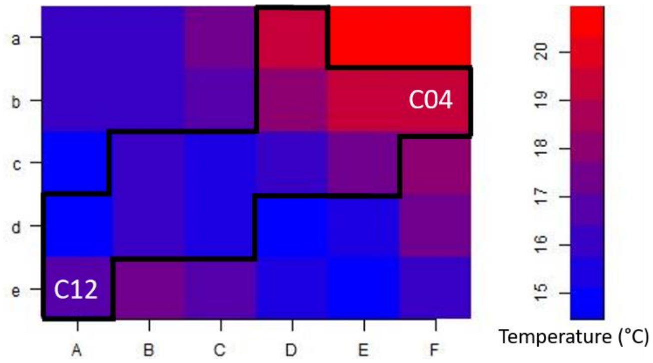

Figure S2: Distribution of temperature in °C across the study area based on the ERA5-Land reanalysis data, which is available at an 11.1 km resolution. Shown is the average temperature from 1991 to 2020, which ranges from 14°C to 21°C. The black line marks the grid cells where coffee farms in our study area are located. The seasonal changes in temperature of two grid cells (C04 and C12) are illustrated in Fig. S6 and Fig. S7.

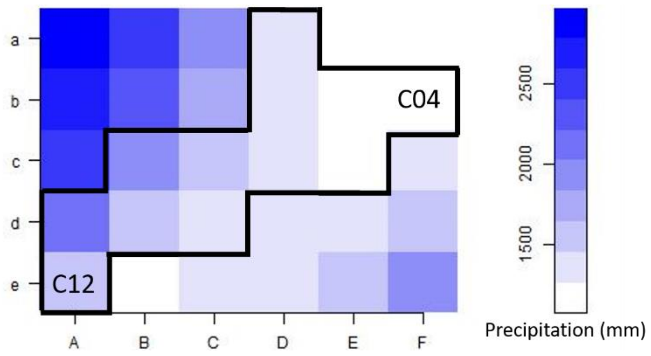

Figure S3: Distribution of precipitation in millimeters along the study area based on the ERA5-Land reanalysis data, which is available at an 11.1 km resolution. Shown is the average value from 1991 to 2020, which ranges from 1000 mm to 3000 mm. The black line marks the grid cells where coffee farms in our study area are located. The seasonal changes in precipitation of two grid cells (C04 and C12) are illustrated in Fig. S6 and Fig. S7.

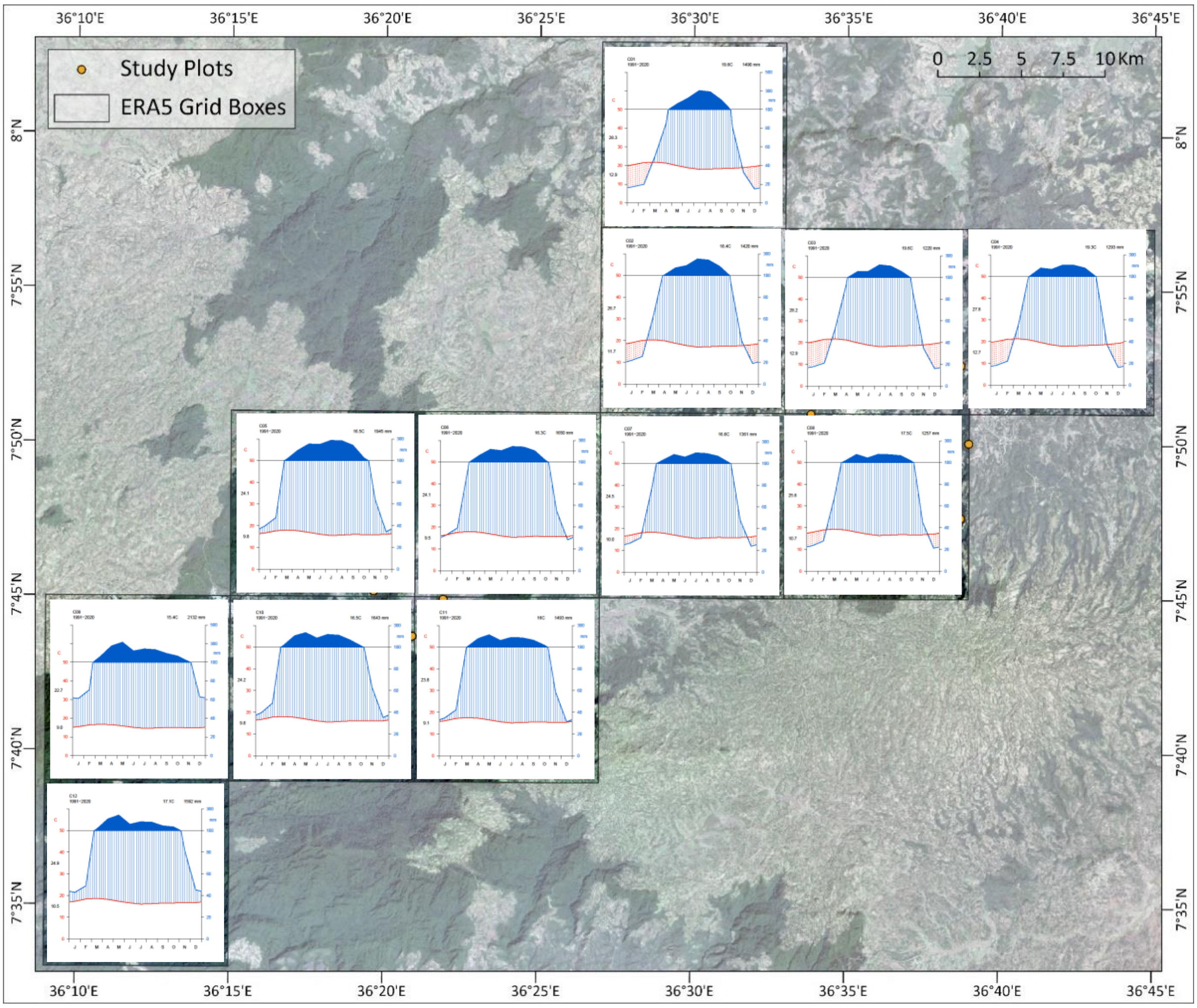

Figure S4: Walter diagrams along the study area in the Jimma zone in southwestern Ethiopia. Each individual Walter diagram shows the seasonal variation in temperature and precipitation averaged across the time period from 1991-2020 and allows a good illustration and categorization of different moisture conditions (dry, humid, wet) (Walter et al., 1975). The diagrams show the average precipitation (blue line) and temperature (red line) as well as the drier conditions (red dots), humid conditions (blue shaded) and wet conditions (blue filled) for each grid cell of the study area.

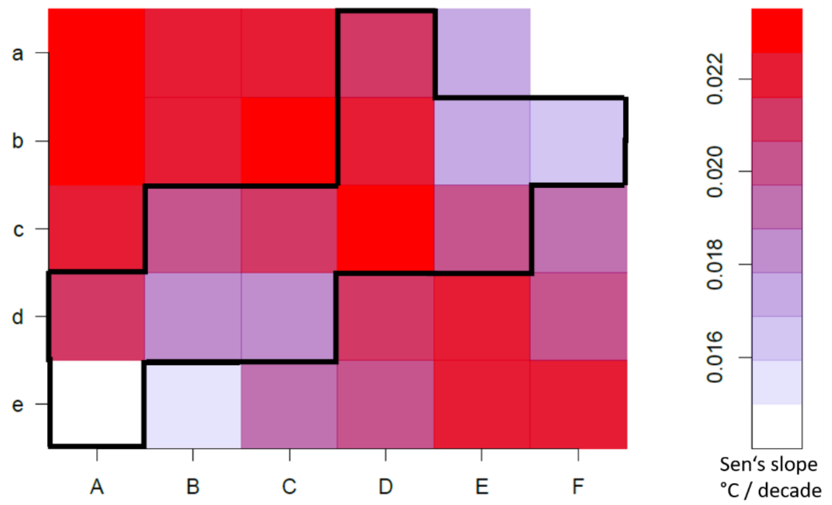

Figure S5: Distribution of temperature gradient in °C per decade along the study area based on the ERA5-Land reanalysis data, which is available at an 11.1 km resolution. Shown is the average value from 1991 to 2020, which ranges from 0.015 °C/decade to 0.022 °C/decade. The black line marks the grid cells where coffee farms in our study area are located.

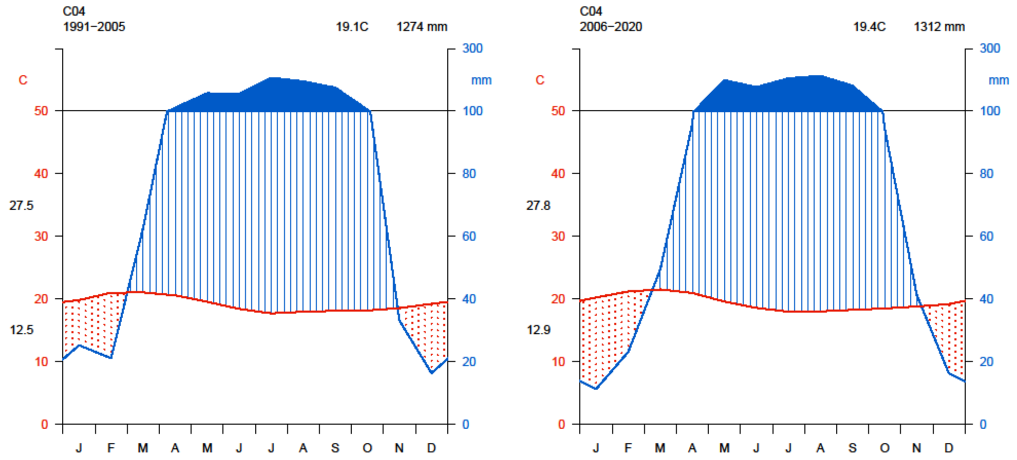

Figure S6: Walter diagram for grid cell C04 of the ERA5-Land dataset showing the average precipitation (blue line) and temperature (red line) for the mean of two different time periods (a) 1991-2005 and (b) 2006-2020.

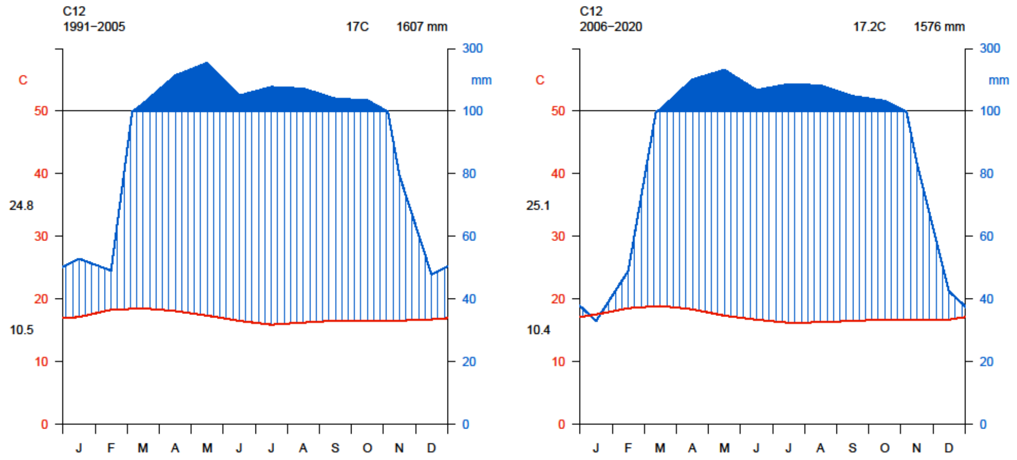

Figure S7: Walter diagram for grid cell C12 of the ERA5-Land dataset showing the average precipitation (blue line) and temperature (red line) for the mean of two different time periods (a) 1991-2005 and (b) 2006-2020.

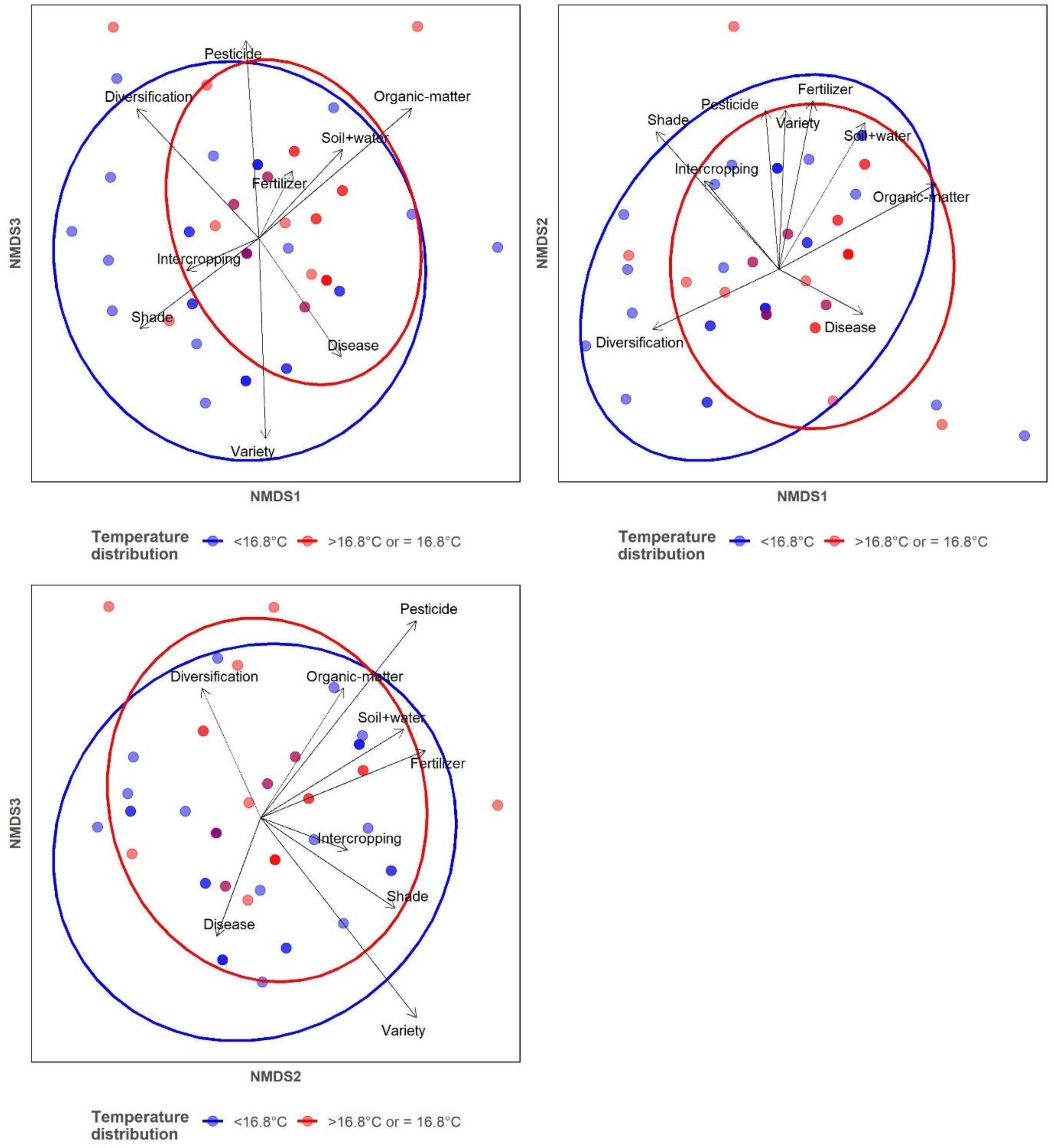

Figure S8. Relationship between the composition of management practices and temperature distribution. The vector names represent the different management practices. The circles indicate the temperature distribution below (blue) and above (red) the mean temperature across all study sites of 16.8°C. The stress value is 0.102 (Clarke, 1993).

Table S1. Statistical overview of the relationships between perceived changes in climatic variables during the last 30 years and spatial variation in local climatic variables in Jimma zone in southwestern Ethiopia. Shown are the odds ratio (OR, with 1°C, 100 mm and 0.01 in SPEI as one unit, respectively), p-value, R^2-value and number of observations. Values are obtained from generalized linear models with a binary distribution and logit link.

**Farmers' perception of changes in:**	**Local climatic variable**	**Odds Ratio**	**p-value**	R^2	***N***
Dry season temperature	Mean annual temperature	1.026	0.919	0.000	56
Rainy season temperature	Mean annual temperature	1.435	0.182	0.049	55
Length of hot spells	Mean annual temperature	1.113	0.673	0.005	55
Length of cold spells	Mean annual temperature	0.607	0.066	0.094	56
Frequency of frost / cold nights	Mean annual temperature	0.983	0.940	0.000	54
Frequency of drought	Mean annual temperature	3.136	0.000	0.408	56
Quantity of rainfall during rainy season	Annual precipitation	1.144	0.188	0.045	55
Length of rainy season	Annual precipitation	1.235	0.034	0.117	54
Length of dry spells during rainy season	Annual precipitation	0.704	0.035	0.187	55
Unseasonal rain in the dry season	Annual precipitation	1.503	0.016	0.218	56
Intensity of rain	Annual precipitation	0.931	0.482	0.013	55
Frequency of drought	Annual precipitation	0.775	0.068	0.11	56
Unseasonal rain in the dry season	SPEI	1.297	0.096	0.078	56
Dry season temperature	SPEI	0.986	0.920	0.000	56
Length of hot spells	SPEI	0.892	0.422	0.017	56
Length of cold spells	SPEI	1.310	0.054	0.095	56
Frequency of frost / cold nights	SPEI	1.134	0.349	0.023	56
Frequency of drought	SPEI	0.510	0.002	0.342	56

Table S2. Statistical overview of the relationships between perceived changes in climatic variables during the last 30 years and spatial variation in the rate of climate change in Jimma zone in southwestern Ethiopia. (OR, with 1°C, 100 mm and 0.01 in SPEI as one unit, respectively), p-value, R^2-value and number of observations. Values are obtained from generalized linear models with a binary distribution and logit link. The rate of climate change is expressed as the Sen's slope.

Farmers' perception of changes in:	Rate of temperature change	Odds Ratio	p-value	R^2	*N*
Dry season temperature	Sen's slope of temperature	0.967	0.839	0.001	56
Rainy season temperature	Sen's slope of temperature	1.015	0.919	0.000	55
Length of hot spells	Sen's slope of temperature	1.069	0.669	0.005	55
Length of cold spells	Sen's slope of temperature	1.149	0.368	0.021	56
Frequency of frost / cold night	Sen's slope of temperature	0.845	0.246	0.034	54
Frequency of drought	Sen's slope of temperature	0.813	0.206	0.044	55
Unseasonal rain in the dry season	Sen's slope of drought severity	1.790	0.256	0.075	56
Dry season temperature	Sen's slope of drought severity	0.971	0.885	0.001	56
Length of hot spells	Sen's slope of drought severity	0.929	0.716	0.003	56
Length of cold spells	Sen's slope of drought severity	1.166	0.392	0.018	56
Frequency of frost / cold night	Sen's slope of drought severity	1.247	0.225	0.037	56
Frequency of drought	Sen's slope of drought severity	0.176	0.403	0.018	56

Table S3. Current SPEI index, and rate of change in the SPEI index, for each of the grid cells in the study area. SPEI values shown are the average monthly SPEI values for the dry period from November to April from 1991-2020, separately for each grid cell in the study area in Jimma zone in southwestern Ethiopia. The Sen's slope represents the magnitude of change in SPEI for the period of 1991-2020, with the significance level calculated by the Mann-Kendall trend test (*** $p < 0.01$, ** $p < 0.05$, * $p < 0.1$).

Grid cell	Average SPEI value during dry period from 1991-2020	Sen's slope
C01	-0.208	-0.020**
C02	-0.191	-0.021***
C03	-0.203	-0.019***
C04	-0.181	-0.018**
C05	-0.147	-0.022**
C06	-0.158	-0.023**
C07	-0.174	-0.024**
C08	-0.180	-0.022***
C09	-0.124	-0.023
C10	-0.144	-0.019
C11	-0.152	-0.023*
C12	-0.141	-0.027*

*** $p < 0.01$, ** $p < 0.05$, * $p < 0.1$

Table S4: Permutational multivariate analysis of variance (PERMANOVA) on the composition of management practices as a function of spatial variation in temperature and precipitation as well as farmers' perceptions of changes in temperature and precipitation since 1991. The model was run separately for each explanatory variable. PERMANOVAs were based on the binomial deviance dissimilarity measure. P-values were obtained using 999 permutations.

**Explanatory variable**	**N**	**Df**	R^2	***P***
**Spatial variation in climate**				
Temperature	53	1	0.050	0.008
Precipitation	53	1	0.031	0.123
SPEI	52	1	0.038	0.066
**Spatial variation in the perception of climate change**				
Perception of temperature changes during dry season	53	1	0.037	0.068
Perception of temperature changes during rainy season	53	1	0.024	0.266
Perception of precipitation changes	49	1	0.004	0.978

References

Clarke, K. R. (1993). Non-parametric multivariate analyses of changes in community structure. *Austral Ecology*, *18*(1), 117–143. https://doi.org/10.1111/j.1442-9993.1993.tb00438.x

Walter, H., Harnickell, E., & Mueller-Dombois, D. (1975). Climate diagram maps. *Ind. Countries and the Ecological Climatic Regions of the Earth. Suppl. To the Veg. Monographs*, *8*(11).
